# Duplex PCR Detection and Differentiation of Insect DNA *Tenebrio molitor* and *Zophobas morio* in Various Types of Food

**DOI:** 10.3390/insects16090983

**Published:** 2025-09-21

**Authors:** Dagmar Stehlíková, Veronika Müllerová, Anna Adámková, Pavel Beran, Martin Adámek, Vladislav Čurn, Soňa Škrovánková, Jiří Mlček

**Affiliations:** 1 Department of Genetics and Biotechnology, Faculty of Agriculture and Technology, University of South Bohemia in Ceske Budejovice, Na Sadkach 1780, 370 05 Ceske Budejovice, Czech Republic; stehld@fzt.jcu.cz (D.S.); mullev05@fzt.jcu.cz (V.M.); beranp@fzt.jcu.cz (P.B.); curn@fzt.jcu.cz (V.Č.); 2 Department of Food Analysis and Chemistry, Faculty of Technology, Tomas Bata University in Zlin, Vavreckova 5669, 760 01 Zlin, Czech Republic; aadamkova@utb.cz (A.A.); skrovankova@utb.cz (S.Š.); 3 Department of Automation and Control Engineering, Faculty of Applied Informatics, Tomas Bata University in Zlin, Nad Stranemi 4511, 760 05 Zlin, Czech Republic; m2adamek@utb.cz

**Keywords:** edible insects, molecular detection, novel food

## Abstract

Edible insects, particularly *Tenebrio molitor* (Linnaeus) (mealworm) and *Zophobas morio* (Fabricius) (superworm), have drawn increasing attention as alternative protein sources. This study aims to develop an accurate molecular detection method for *T. molitor*, an EU-approved food species, and to differentiate it from *Z. morio*, which remains unapproved for human consumption in the EU. The process enables precise and sensitive identification methods by optimizing singleplex and duplex PCR techniques targeting 16S rRNA and COI gene regions. The DNA of *T. molitor* was detected in various food matrices, including pastries, chocolate, and porridge, while avoiding cross-reactivity with *Z. morio*, *Gryllus asimilis*, and *Locusta migratoria*. The detection limit for both singleplex and duplex PCR was 10 pg of DNA, ensuring robustness against inhibitory effects from complex food matrices. The developed approach ensures reliable detection and compliance with EU regulations regarding insect-based foods, providing a critical tool for food authentication and preventing adulteration. The key advancements of this approach lie in its improved specificity and sensitivity, allowing for the ability to detect complex food matrices. An applied perspective was evaluated using real commercial food products.

## Simple Summary

In this study, two types of edible insects were examined: mealworms (*Tenebrio molitor*) and superworms (*Zophobas morio*). While mealworms are approved for use in food in the EU, superworms are not. Therefore, only approved insect species can be used in food products. Duplex PCR was developed to detect whether a food product contains mealworms and to ensure that superworms are not included. This method was designed to be highly accurate, even when applied to complex foods such as bread, chocolate, or porridge. Insect DNA was successfully detected, even in very small amounts. Various food products were tested. All of them were labeled as containing mealworms, and this was confirmed through testing. No traces of superworms were found. Even after cooking or baking, the insects could still be identified using the method. Using this approach, food fraud can be prevented, consumer protection can be improved, and EU food regulations can be followed more effectively. The control of insect ingredients in food products, therefore, will be easier.

## 1. Introduction

The demand for food is driven not only by the growing human population, but also by rising living standards. Therefore, the production of proteins from sustainable systems is a highly debated topic. Proteins obtained from non-conventional sources must be safe, nutritionally valuable, produced in sufficient and stable quantities, and be accepted by consumers. Edible insects, one of the non-conventional commodities, have high potential as an alternative protein source, and could become an important food and feed raw material [1]. Additionally, insects may also offer a more efficient way of producing animal protein with respect to environmental sustainability and the use of cost-effective raw materials [2,3,4].

As a reaction to rising interest in using insects as food, some species from orders Coleoptera and Orthoptera were added to the European Union’s novel food list. One of them, *T. molitor*, or the yellow mealworm, used to be solely viewed as a storage pest and pet feed. In 2021, *T. molitor* larvae were marked as safe for consumption by the EU [5]. Recently, foodstuffs containing *T. molitor* larvae have entered the European food market, with the aim of increasing the protein content in products while utilizing a promising alternative protein source. Dried larvae are sold as whole in different flavors, as well as in the form of meal incorporated in pastry, chocolate, porridge, or snack bars. Raising *T. molitor* as a protein source is attractive due to lower space requirements, easy maintenance, high yield, and relatively low price [6].

For safe consumption, control measures need to be established in order to monitor the correct use of product packaging and to detect fraudulent activity. For confirming *T. molitor* content in insect-based foods, a method is needed to differentiate other similar species, which are not on the novel foods list. Confusion in detection could be caused by *Z. morio*, also called superworm. *Z. morio* is also a storage pest belonging to the same family Tenebrionidae. The larger size of *Z. morio* larvae and their lower maintenance costs could lead to potential implementation in insect-based foods. However, *Z. morio* larvae are currently not approved for consumption in the EU, and remain used only as animal feed. Using a modified microscopic method incorporating a double-sedimentation protocol, insect particles were detected in poultry feed containing 1% insect material, including *T. molitor*. While fragments of muscle tissue and tracheoles confirmed the presence of insect-derived material, they did not allow for species-level identification. Therefore, a method to verify the content of approved insect species in insect-based food is needed [7]. There are no validated immunoassays with antibodies specific for *T. molitor* yet. However, there is a more general pilot use of standard ELISA kits (e.g., for crustacean tropomyosin or chitosan detection) [8,9].

Molecular methods pose a reliable approach to insect detection and identification in foodstuffs. Polymerase chain reaction stands out as a reference method due to its sensitivity and accuracy [10]. Nonetheless, the accuracy of this method could be influenced by input material, as certain food ingredients could inhibit the PCR reaction and cause inaccurate outcomes. Thus, optimization of the method is necessary. This study aims to propose a singleplex and multiplex PCR method to verify *T. molitor* content in insect-based foods sold on the Czech food market, such as pastry, chocolate, and porridge. Specificity testing is performed against *Z. morio* and two other novel food insect species, *G. asimilis* and *L. migratoria*. This study addresses the critical need for the reliable identification of approved insect species in food products, focusing on *T. molitor*. Accurate detection is essential for consumer safety and regulatory compliance. This is the first optimized molecular method for the verification of *T. molitor* in processed foods on the Czech market, making this work a new and significant contribution to the field.

## 2. Materials and Methods

### 2.1. Insect Sample Preparation

Prior to the analysis, larvae of *T. molitor*, *Z. morio* in the last and second-to-last instars of development (full body length just before pupation), and imagos of *L. migratoria* and *G. assimilis* were collected in the insectarium of UTB Zlín and transferred to the laboratory. The samples, both larvae and imagos, were starved for 48 h, killed with boiling water (100 °C), and dried at 105 °C. Subsequently, the samples were milled in a bead-beater, homogenized, and stored in a refrigerator at 4–7 °C until analysis.

### 2.2. Pastry Samples

Pastry bread containing different percentages of edible insect larvae meal were prepared for the detection of edible insect species in foodstuffs. The bread dough was composed of 55 mL water, 0.93 g sugar, 0.75 g salt, 0.9 g yeast, and 50 g mix of white flour and different amounts of larvae meal (1%, 2.5%, 10%). Finally, the doughs were baked in a hot-air oven for 35 min at 165 °C.

### 2.3. Insect-Based Food

In addition to pastry, different insect-based foods were used for analysis. All products are sold on the Czech food market and contain *T. molitor* larvae. Selection of products included samples of dried larvae, “Křupaví červíci” (WormUP, Blansko, Czech Republic), with garlic and chili flavors and dark low-carb chocolate (Farmerio, Svatoňovice, Czech Republic) containing cocoa beans, cocoa butter, and *T. molitor* larvae. The last analyzed product was instant rice porridge (Farmerio, Svatoňovice, Czech Republic), containing husked rice (84%), hominy (2.5%), *T. molitor* larvae meal (11.5%), and lyophilized raspberries (2%).

### 2.4. DNA Extraction

In total, 30 mg of material was homogenized using 3 mm metal beads in a Retsch^®^ Mixer Mill MM400 (Haan, Germany) for 30 s at a maximal frequency (30 Hz). The DNA was extracted by a modified CTAB PVP method [11]. Finally, the DNA pellet was dissolved in 1× TE buffer and stored in −20 °C. The quality and quantity of the extracted DNA were assessed spectrophotometrically using a BioSpec-nano spectrophotometer (Shimadzu, Long Beach, CA, USA) by measuring the absorbance at 260 nm (A260) and 280 nm (A280). DNA purity was determined by calculating the A260/A280 ratio.

### 2.5. PCR Analysis

The tested primers were designed to target specific regions of the 16S rRNA and COI genes of *T. molitor* and *Z. morio*, respectively [11]. We tested their coamplification in a novel multiplex PCR by modifying the PCR protocol with primers TM_F (5′ CAAAGGTAGCATAATCATTAGT 3′), TM_R (5′ AGTTAAATAAATTTTCTAACCG 3′), ZM_F (5′ GGGCATCAGTCGATCTCGCA 3′), and ZM_R (5′ CGATCAAAAGTTATTCCTTGTGG 3′) [12]. PCR amplification was performed using a Biometra TProfessional thermocycler (Biometra GmbH, Göttingen, Germany). To optimize annealing temperature, a gradient PCR was conducted (52–60 °C) for each primer set. The optimal annealing temperature was determined to be 55 °C for both sets of primers. Each 25 μL PCR reaction contained 12.5 μL One Taq^®^ Hot Start Quick-Load^®^ Master Mix (New England Biolabs, Ipswich, MA, USA), 9.5 μL PCR Ultra H_2_O Top-Bio, 1 μL forward primer, 1 μL reverse primer, and 1 μL template DNA. PCR conditions were as follows: initial denaturation at 94 °C for 5 min, followed by 35 cycles of denaturation at 94 °C for 60 s, annealing at 55 °C for 30 s, and extension at 68 °C for 60 s. A final extension step was performed at 68 °C for 5 min. PCR products were analyzed by electrophoresis on a 2% agarose gel in 1× TBE buffer. A 100 bp DNA ladder (Neb, Hitchin, UK) was used as a size marker. Electrophoresis was carried out at 120 V for 40 min, and the gel was visualized using InGenius 3 (SynGene, Cambridge, UK).

### 2.6. Specificity and Sensitivity Testing

The specificity of the PCR detection was verified using DNA from four insect species. Ten nanograms of DNA were used in each PCR reaction, and each DNA extract was tested in duplicate. To confirm the specificity of the primers to their target species, PCR products were extracted from gel using NucleoSpin Gel and PCR Clean-up (Macherey-Nagel, Düren, Germany), sequenced by an external service provider SEQme s.r.o. (Dobris, Czech Republic), and analyzed using BLAST (version 2.15.0; NCBI, Bethesda, MD, USA) [13]. To determine the limit of detection (LOD), 10-fold serial dilutions of known amounts (100 ng/μL) of *T. molitor* and *Z. morio*. DNA were tested using both singleplex and multiplex PCR. The LOD was determined as the lowest concentration of DNA that could be reliably detected.

## 3. Results

### 3.1. Samples

**Figure 1:**
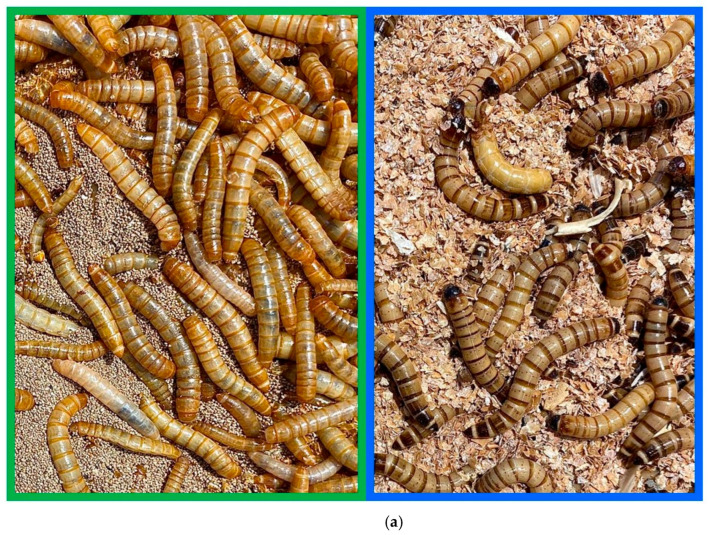

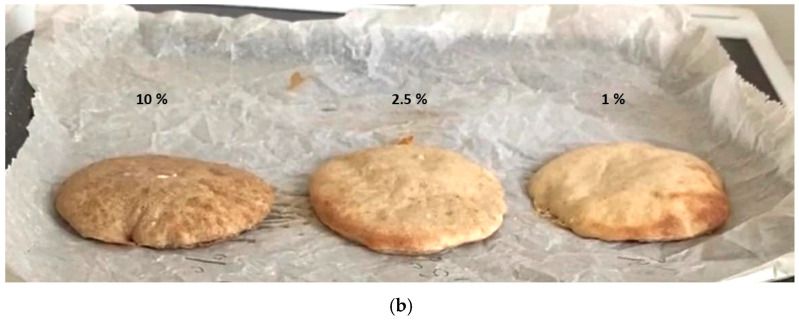
Fresh samples and pastry samples: (**a**) larvae and imagos of *T. molitor* (left green line) *Z. morio* (right blue line) before drying; (**b**) baked bread with different amounts (1%, 2.5%, 10%) of larvae meal (example with *T. molitor*).

Positive controls were prepared milled of dried larvae and imagos of *T. molitor*, *Z. morio* (Figure 1a). Pastry breads were baked with different amounts of larvae meal (1%, 2.5%, 10%) (Figure 1b).

### 3.2. DNA Extraction

DNA concentrations ranged from tens to hundreds of ng/µL. Purity levels were influenced by the type of input material used. At 1% insect meal content, the total nucleic acid concentration was the lowest among the three tested levels. In commercial insect-containing products, the detection efficacy was the lowest; however, no significant PCR inhibition was observed. Among the tested matrices, chocolate exhibited the highest inhibitory effect.

### 3.3. PCR

Singleplex and duplex PCR approaches were used to detect the presence of edible insects in food. In the case of *T. molitor*, a 240 bp fragment of the 16S rRNA gene was specifically amplified, while in the case of *Z. morio*, a 120 bp fragment of the COI gene was amplified. Both primer sets were tested at six annealing temperatures. Temperatures of 52 and 55 °C were suitable for the detection of *T. molitor*, while *Z. morio* were detected at all six tested temperatures.

**Figure 2:**
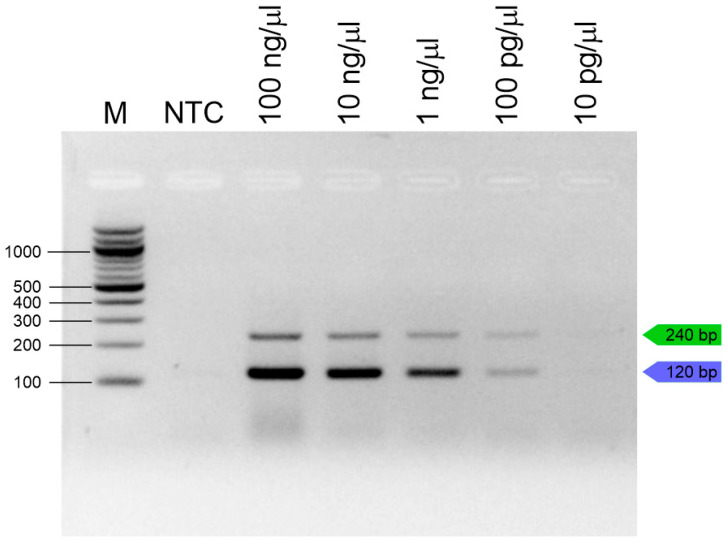
Sensitivity of duplex PCR (10-fold serial dilution); green lines (*T. molitor*); blue lines (*Z. morio*), M—100 bp ladder (NEB, UK); NTC—no-template control.

To determine the sensitivity of the singleplex and duplex PCR assays, DNA isolated from crushed *T. molitor* and *Z. morio* samples was used. Singleplex and duplex PCR were performed using 100 ng–1 pg of DNA as a template and the *T. molitor* and *Z. morio* primers. A DNA concentration of 100 pg was determined as the limit of detection for both singleplex and duplex PCR (Figure 2).

**Figure 3:**
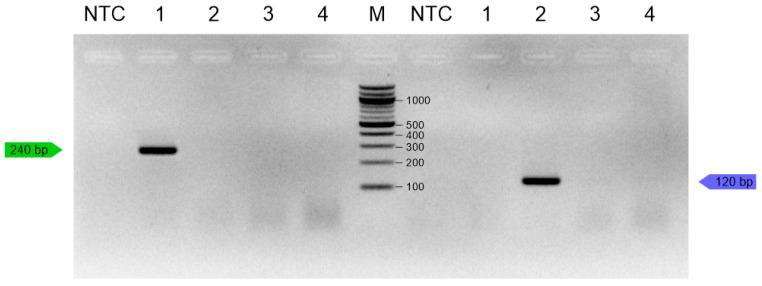
Singleplex specificity in test with 1—*T. molitor* (green line), 2—*Z. morio* (blue line), 3—*G. assimilis*, 4—*L. migratoria*; M—100 bp ladder (NEB, UK); NTC—no-template control.

In duplex PCR, no cross-reactions were observed, and sequencing PCR products showed 100% similarity to *T. molitor* and *Z. morio* sequences in the GenBank database and confirmed the specificity of the primers. Singleplex and duplex PCR accurately and specifically detected insect DNA from the target species. In accordance with EU Regulation 2015/2283, *G. assimilis*, *L. migratoria*, *T. molitor*, and *Z. morio* were selected for specificity testing. Singleplex PCR resulted in a single 240 bp amplicon for *T. molitor* and a single 120 bp amplicon for *Z. morio* (Figure 3).

**Figure 4:**
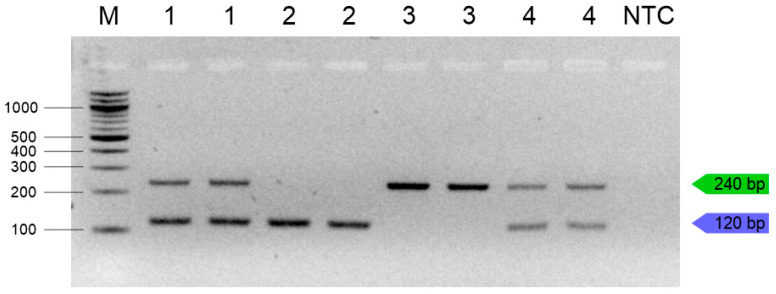
Specificity in multiplex PCR with different combinations DNA samples. 1—0.5 µL DNA *T. molitor* and 0.5 µL *Z. morio*; 2—1 µL *Z. morio*; 3—1 µL *T. molitor*; 4—0.25 µL *Z. morio*, 0.25 µL *T. molitor*, 0.25 µL *G. assimilis*, and 0.25 µL *L. migratoria*; M—100 bp ladder (NEB, UK); NTC—no-template control; *T. molitor* (green line), *Z. morio* (blue line).

Duplex PCR was performed using different amounts of DNA from various insect species. In all of the tested combinations, both *Z. morio* and *T. molitor* could be specifically detected in a single reaction (Figure 4).

### 3.4. Verification

**Figure 5:**
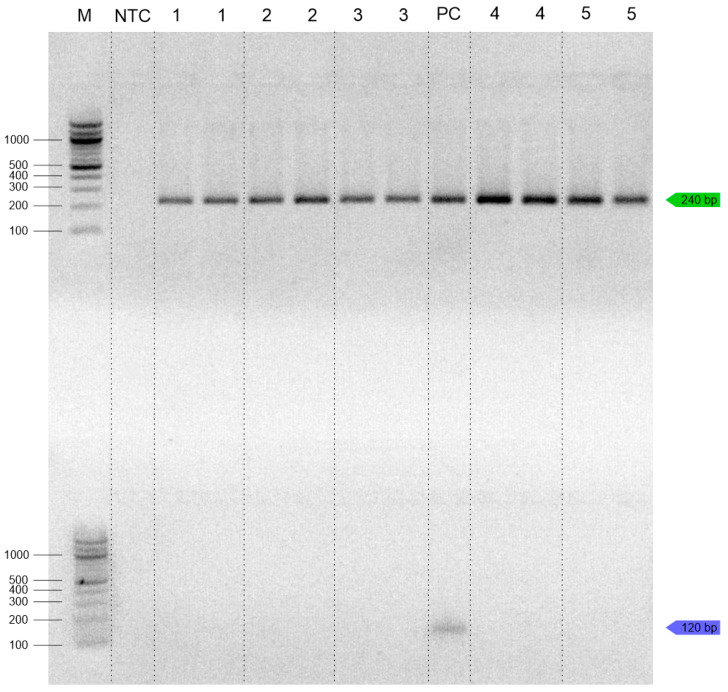
Products with *T. molitor*. 1—prepared boiled instant rice porridge (Farmerio, Svatoňovice, Czech Republic); 2—raw instant rice porridge (Farmerio, Svatoňovice, Czech Republic); 3—low-carb chocolate (Farmerio, Svatoňovice, Czech Republic); 4—dried larvae, “Křupaví červíci” (WormUP, Blansko, Czech Republic) with garlic; 5—dried larvae, “Křupaví červíci” (WormUP, Blansko, Czech Republic), with chili; M—100 bp ladder (NEB, UK); PC—10 ng/µL *T. molitor* (up) and *Z. morio* (down); NTC—no-template control; up (green)—PCR for detection *T. molitor*; down (blue)—PCR for detection *Z. morio*.

For specific confirmation, insect-based foods (declared as containing only *T. molitor*) from the Czech food market were used. All products were tested separately for *T. molitor* and *Z. morio* using PCR. Positive detection of *T. molitor* and negative detection of *Z. morio* were observed in all products (Figure 5). This confirms that companies accurately declare the insect species present in their foods.

**Figure 6:**
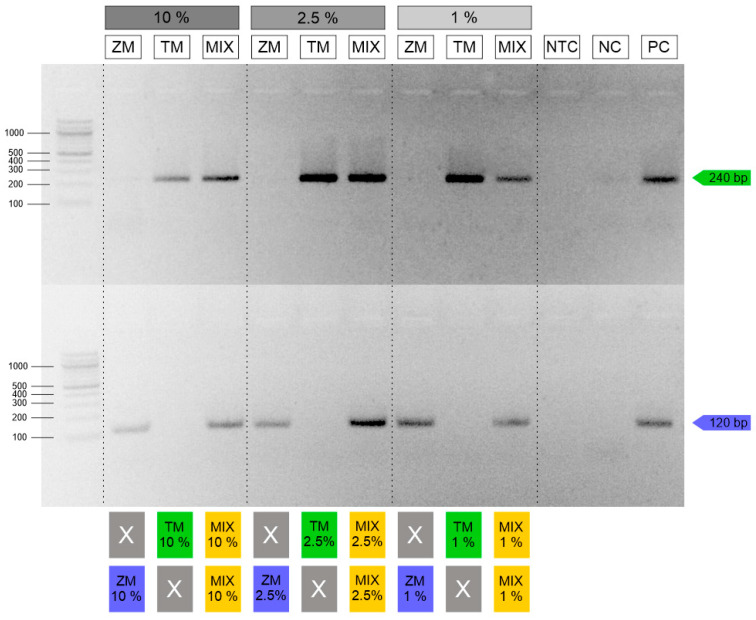
Pastry samples. Up (green) TM—PCR for detection *T. molitor*; down (blue) ZM—PCR for detection *Z. morio*; MIX (yellow)—*T. molitor* and *Z. morio*; PC—10 ng/µL *T. molitor* (up) and *Z. morio* (down); NTC—no-template control; X—not detected.

In pastry breads containing 1–10% insects, detection of the insect content is possible. The baking process did not affect the detection compared to raw insects at these percentages. In baked bread containing a combination of *T. molitor* and *Z. morio*, individual insect species can be specifically detected at levels as low as 1% insect content. This represents a very promising method for identifying counterfeit products (Figure 6).

## 4. Discussion

Edible insects have recently been used as snacks in Western Europe and as food additives to improve product texture. Fortifying dough with insect meal can alter both its nutritional and rheological properties. It was demonstrated that the substitution of starch with cricket meal in gluten-free baked goods reduced bread hardness and improved dough consistency by enhancing water binding [14]. Similarly, it was found that adding 5% cricket meal to wheat bread dough resulted in higher bread volume and lower crumb stiffness [15].

The rheological properties of gluten-free doughs are generally more complex than those of gluten-containing doughs. Gluten provides the desired baking qualities, such as viscosity and elasticity [16]. However, these properties are suppressed in gluten-free flours due to the absence of gluten, and efforts are made to use improving agents to achieve higher quality and more consumer-acceptable products.

Improving agents can serve various purposes, including speeding up proofing, increasing the volume of products, enhancing elasticity and workability, prolonging shelf life, and reducing production time [17,18]. Edible insect meal can potentially improve the rheological properties of dough, especially gluten-free dough, but the properties may vary depending on the insect species used. Differences in species may also affect price and quality, potentially leading to the substitution (adulteration) of higher-quality insects with cheaper, lower-quality ones. In recent years, instances of fraud have been identified within the edible insect supply chain [5,19].

The legislative process is relatively simpler in Asian countries, while in the EU, only four insect species can be used for human consumption: yellow mealworm (*T. molitor*), migratory locust/grasshopper (*L. migratoria*), grain mould beetle (*Alphitobius diaperinus*), and house cricket (*Acheta domesticus*). These insects are undergoing risk and food safety assessments for the protection of public health by EFSA. One of the safety controls is the assessment of the product’s nutritional value, particularly protein content [20]. Different insects can trigger the development of clinical symptoms typical of allergenic reactions in sensitized individuals. This is the next reason why specific detection methods are necessary [21].

Mitochondrial genome analysis was one of the earliest molecular biological methods applied to insect studies. The ribosomal RNA gene (16s and 12s rRNA), NADH5 and NADH4 dehydrogenase gene, and cytochrome c oxidase gene were used for designing specific insect primers [22,23]. Afterward, these genes were used for sequenced insects and new population studies [24,25].

While several modified PCR methods have been described for *T. molitor* detection, cross-amplification remains a challenge. For example, primers targeting the Cadherin gene have been shown to produce false positives for *Z. morio*, *Bombyx mori*, and *Gammarus* sp. Primers targeting the Wingless gene offer improved specificity, but can still cross-react with plant DNA, particularly wheat, a common ingredient in insect-based food and feed products [10,26].

However, existing studies demonstrate the feasibility of PCR for the detection of several edible insect species, but did not address the critical issue of differentiating *T. molitor* from closely related species. Our work provides several novel and practically relevant contributions. First, the duplex PCR developed in this study achieved a detection limit of 100 pg of DNA, confirming high analytical sensitivity suitable for complex food matrices. Second, specificity was rigorously validated not only against *Z. morio*, but also against other insect species relevant to the European novel food context, namely *G. assimilis* and *L. migratoria*. This broader specificity testing minimizes the risk of cross-reactivity and strengthens the robustness of the method. Most importantly, from an applied perspective, the assay was evaluated on real commercial food products, including chocolate, porridge, and flavored dried larvae. This step bridges the gap between laboratory validation and application, underscoring the method’s utility for food control authorities [12,27,28]. According to EU Regulation 2015/2283, specific tests are needed to distinguish between *Z. morio* and *T. molitor* in insect-based food. In this study, we used primers that were previously shown to be highly sensitive and specific in separate PCR detections. This study introduces a multiplex detection method capable of identifying low-level targets, aiming to enhance food product safety and quality control.

## 5. Conclusions

The multiplex PCR method described in this study is robust and sensitive, making it suitable for its intended purpose: the specific detection of edible insects in insect-based food. The two primer pairs enabled the discrimination of *T. molitor* and *Z. morio*. The sensitivity and specificity of the method were confirmed by the robust detection of target DNA. While inhibitors present in the food or insect matrix can potentially affect DNA extraction, the developed methods were robust and consistently detected *T. molitor* and *Z. morio* target DNA. Further testing on processed foods under more extreme conditions is necessary to support routine food control applications. For these reasons, the method is suitable for detecting the presence/absence of *T. molitor* and *Z. morio* DNA in food, even in small amounts, as well as in insect-based food products.

## Data Availability

The data presented in this study are available on request from the corresponding author. The data are not publicly available due to privacy restrictions.

## References

[1] Mlček J., Adámková A., Adámek M., Borkovcová M., Bednářová M., Kouřimská L., Hlobilová V. (2021). Selected aspects of edible insect rearing and consumption—A review. Czech J. Food Sci..

[2] Premalatha M., Abbasi T., Abbasi T., Abbasi S.A. (2011). Energy-efficient food production to reduce global warming and ecodegradation: The use of edible insects. Renew. Sustain. Energy Rev..

[3] Pimentel D., Pimentel M. (2003). Sustainability of meat-based and plant-based diets and the environment. Am. J. Clin. Nutr..

[4] Oonincx D.G., De Boer I.J. (2012). Environmental impact of the production of mealworms as a protein source for humans–a life cycle assessment. PLoS ONE.

[5] Traynor A., Burns D.T., Wu D., Karoonuthaisiri N., Petchkongkaew A., Elliott C.T. (2024). An analysis of emerging food safety and fraud risks of novel insect proteins within complex supply chains. npj Sci. Food.

[6] Siemianowska E., Kosewska A., Aljewicz M., Skibniewska K.A., Polak-Juszczak L., Jarocki A., Jedras M. (2013). Larvae of mealworm (*Tenebrio molitor* L.) as European novel food. Agric. Sci..

[7] Weiner A., Kwiatek K. (2002). Laboratory experience with the microscopic method for the detection of insects in poultry feeds. J. Vet. Res..

[8] Delgado Calvo-Flores L., Garino C., Moreno F.J., Broll H. (2022). Insects in food and their relevance regarding allergenicity assessment. EFSA J..

[9] Pospiech M., Pečová M., Bartlová M., Javůrková Z., Kopecká A., Šebelová K., Hajšlová J. (2024). Development of Indirect Sandwich ELLA for Detection of Insects in Food. Appl. Sci..

[10] Debode F., Marien A., Gérard A., Francis F., Fumière O., Berben G. (2017). Development of real-time PCR tests for the detection of *Tenebrio molitor* in food and feed. Food Addit. Contam..

[11] Doyle J., Hewitt G.M., Johnston A.W., Young J.P.W. (1991). DNA protocols for plants. Molecular Techniques in Taxonomy.

[12] Tramuta C., Gallina S., Bellio A., Bianchi D.M., Chiesa F., Rubiola S., Romano A., Decastelli L. (2018). A set of multiplex polymerase chain reactions for genomic detection of nine edible insect species in foods. J. Insect Sci..

[13] Altschul S.F., Gish W., Miller W., Myers E.W., Lipman D.J. (1990). Basic local alignment search tool. J. Mol. Biol..

[14] Kowalczewski P.Ł., Walkowiak K., Masewicz Ł., Bartczak O., Lewandowicz J., Kubiak P., Baranowska H.M. (2019). Gluten-free bread with cricket powder—Mechanical properties and molecular water dynamics in dough and ready product. Foods.

[15] Roncolini A., Milanović V., Cardinali F., Osimani A., Garofalo C., Sabbatini R., Clementi F., Pasquini M., Mozzon M., Foligni R. (2019). Protein fortification with mealworm (*Tenebrio molitor* L.) powder: Effect on textural, microbiological, nutritional and sensory features of bread. PLoS ONE.

[16] Cappelli A., Oliva N., Cini E. (2020). A systematic review of gluten-free dough and bread: Dough rheology, bread characteristics, and improvement strategies. Appl. Sci..

[17] Mondal A., Datta A.K. (2008). Bread baking—A review. J. Food Eng..

[18] Burešová I., Kráčmar S., Dvořáková P., Středa T. (2014). The relationship between rheological characteristics of gluten-free dough and the quality of biologically leavened bread. J. Cereal Sci..

[19] Moore J.C., Spink J., Lipp M. (2012). Development and application of a database of food ingredient fraud and economically motivated adulteration from 1980 to 2010. J. Food Sci..

[20] EFSA NDA Panel (2021). Safety of dried yellow mealworm (*Tenebrio molitor* larva) as a novel food pursuant to Regulation (EU) 2015/2283. EFSA J..

[21] Garino C., Zagon J., Braeuning A., German Federal Institute for Risk Assessment (BfR), National Reference Laboratory for Animal protein in Feed, NRL-AP (2019). Insects in food and feed–allergenicity risk assessment and analytical detection. EFSA J..

[22] Kambhampati S., Smith P.T. (1995). PCR primers for the amplification of four insect mitochondrial gene fragments. Insect Mol. Biol..

[23] Ji Y.J., Zhang D.X., He L.J. (2003). Evolutionary conservation and versatility of a new set of primers for amplifying the ribosomal internal transcribed spacer regions in insects and other invertebrates. Mol. Ecol. Notes.

[24] Pons J., Barraclough T.G., Gomez-Zurita J., Cardoso A., Duran D.P., Hazell S., Kamoun S., Sumlin W.D., Vogler A.P. (2006). Sequence-based species delimitation for the DNA taxonomy of undescribed insects. Syst. Biol..

[25] Hillinger S., Saeckler J., Domig K.J., Dobrovolny S., Hochegger R. (2023). Development of a DNA Metabarcoding Method for the Identification of Insects in Food. Foods.

[26] Natonek-Wiśniewska M., Krzyścin P., Koseniuk A. (2022). Qualitative and Quantitative Detection of Mealworm DNA in Raw and Commercial Food Products Using Real-Time PCR. Genes.

[27] Köppel R., Schum R., Habermacher M., Sester C., Piller L.E., Meissner S., Pietsch K. (2019). Multiplex real-time PCR for the detection of insect DNA and determination of contents of *Tenebrio molitor*, *Locusta migratoria* and *Achaeta domestica* in food. Eur. Food Res. Technol..

[28] Villa C., Moura M.B., Teixeira C.S., Costa J., Mafra I. (2023). Monitoring Yellow Mealworm (*Tenebrio molitor*) as a Potential Novel Allergenic Food: Effect of Food Processing and Matrix. Nutrients.

